# Vasospasm-Related Death after Aneurysmal Subarachnoid Hemorrhage: A Retrospective Case–Control Study

**DOI:** 10.3390/jcm11164642

**Published:** 2022-08-09

**Authors:** Ali Khanafer, Pervinder Bhogal, Victoria Hellstern, Christoph Harmening, Hansjörg Bäzner, Oliver Ganslandt, Hans Henkes

**Affiliations:** 1 Neuroradiological Clinic, Klinikum Stuttgart, D-70174 Stuttgart, Germany; 2 Interventional Neuroradiology Department, The Royal London Hospital, Barts NHS Trust, London E1 1FR, UK; 3 Clinic for Anesthesiology and Surgical Intensive Care Medicine, Klinikum Stuttgart, D-70174 Stuttgart, Germany; 4 Neurological Clinic, Klinikum Stuttgart, D-70174 Stuttgart, Germany; 5 Neurosurgical Clinic, Klinikum Stuttgart, D-70174 Stuttgart, Germany; 6 Medical Faculty, University Duisburg-Essen, D-47057 Essen, Germany

**Keywords:** cerebral vasospasm, endovascular treatment, intracranial aneurysm, subarachnoid hemorrhage

## Abstract

Background: Vasospasm after the rupture of an intracranial aneurysm is a frequent phenomenon and is the main cause of morbidity and mortality in patients who have survived intracranial hemorrhage and aneurysm treatment. We analyzed the diagnosis and management of patients with aneurysmal subarachnoid hemorrhage who eventually died from ischemic brain damage due to vasospasm. Methods: Between January 2007 and December 2021 (15 years), a total of 1064 patients were diagnosed with an aneurysmal intracranial hemorrhage in a single comprehensive neurovascular center. Vasospasm was diagnosed in 408 patients (38.4%). A total of 187 patients (17.6%) died within 90 days of the aneurysm rupture. In 64 of these 187 patients (33.7%), vasospasm was considered to be the cause of death. In a retrospective analysis, demographic and clinical data for patients without, with non-fatal, and with fatal vasospasm were compared. The patients with fatal vasospasm were categorized into the following subgroups: “no diagnosis and treatment” (Group a), “delayed diagnosis” (Group b), “cardiovascular complications” (Group c), and “vasospasm-treatment complications” (Group d). Results: Among the patients with fatal vasospasm, 31 (48.4%) were assigned to group a, 26 (40.6%) to group b, seven (10.9%) to group c, and none (0%) to group d. Conclusion: The early recognition of severe posthemorrhagic vasospasm is a prerequisite for any treatment and requires routine diagnostic imaging in all unconscious patients. Aggressive endovascular vasospasm treatment may fail to prevent death but is infrequently the cause of a fatal outcome.

## 1. Introduction

Annually, approximately 10 adults per 100,000 in Europe and the USA experience aneurysmal subarachnoid hemorrhage (aSAH) [1]. The morbidity and mortality of this condition are 40% and 35%, respectively [2]. Both the microsurgical and the endovascular treatment (EVT) of ruptured aneurysms have improved considerably during the last 30 years, despite variations in the techniques and timings [3,4,5].

Posthemorrhagic vasospasm is a pathological constriction of the intracranial arteries, triggered by blood in the subarachnoid space. Radiographic evidence of vasospasm is evident in 50% to 70% of patients with aSAH, about 30% of whom exhibit neurological deficits [6,7].

The incidences of morbidity and mortality due to vasospasm are both around 20% [8,9]. Vasospasm can be diagnosed by transcranial Doppler (TCD) ultrasonography and transcranial color-coded Doppler (TCCD) ultrasonography, albeit with limited accuracy [10]. More reliable methods that are associated with significant logistic efforts are computed tomography angiography (CTA), computed tomography perfusion (CTP) [11], and magnetic resonance angiography (MRA) [12]. The optimal treatment for post-hemorrhagic vasospasm remains unclear. The results achieved with intravenous (IV) or *per os* (PO) administration of calcium-channel blockers have been contentious [13]. EVT strategies include the intra-arterial (IA) administration of nimodipine [14], milrinone [15,16], and verapamil [17]. The long-term IA selective infusion of nimodipine has proved relatively useful [18]. For vasospasm of large arteries, mechanical vessel dilatation with non-compliant balloons, compliant balloons, [19,20,21,22,23,24], and stent-retrievers [25,26,27,28,29] has been advocated.

In this context, the scenarios resulting in a fatal outcome due to vasospasm after aSAH are poorly understood. Posthemorrhagic vasospasm is commonly viewed as fateful. Here, we retrospectively analyzed the case histories of patients who had died from posthemorrhagic vasospasm with the aim of developing a proactive regimen of diagnosis and treatment for this serious condition.

## 2. Materials and Methods

### 2.1. Study Population

Between January 2007 and December 2021, a total of 1064 patients were diagnosed with aSAH in a single neurovascular center. For all patients, clinical data and the results of computed tomography (CT), digital subtraction angiography (DSA), and magnetic resonance imaging (MRI) were evaluated retrospectively.

### 2.2. General Management Strategy

During the 15-year study period, consistent management strategies were followed. These included: early diagnosis of aSAH based on CT/CTA or MRI/MRA and, if necessary, cerebrospinal fluid (CSF) analysis; early external CSF drainage, if considered necessary based on imaging results; early aneurysm treatment with a coil-first policy or microsurgical clipping based on individual interdisciplinary decision-making; and early IV administration of nimodipine.

The initial diagnosis of vasospasm was primarily based on TCD and TCCD. TCD and TCCD were performed according to the same protocol by the attending neurologists or neurosurgeons in the intensive care units. Mean flow velocity (MVF) was determined at least once daily in all brain regions in A1, A2, M1, M2, P1, basilar artery (BA), and V4 segments on both sides. An MFV of >120 cm/s was classified as incipient vasospasms with a potential for a severe course. Therefore, other diagnostic tests were required to confirm or rule out the results of the TCD finding.

No formal standard operating procedures (SOPs) for CTA/CTP, MRI/MRA, or DSA were in place. The diagnosis of vasospasm was made in the affected patients, whether or not they died from vasospasm, after confirmation by diagnostic imaging (CTA, MRA, or DSA) either in terms of routine control or after suspicion of vasospasm by daily TCD control with an MVF of >120. All imaging modalities were, however, available on a 24/7 basis.

### 2.3. Data Analysis

All 1064 data sets were analyzed individually. Vasospasm was either prospectively diagnosed during the hospital stay or retrospectively identified through analysis of the imaging material by a neuroradiologist with four years of professional experience, supervised by the senior author (hh).

Patients were categorized as follows according to the occurrence and severity of vasospasm: Group 1, no vasospasm; Group 2, non-fatal vasospasm; and Group 3, fatal vasospasm. The details of the vasospasm treatment in Group 2 were beyond the scope of this study and are not considered further here. Groups 1, 2, and 3 were compared for demographic and basic clinical data. 

The patients in Group 3 had high-grade vasospasm, causing extensive ischemia in multiple arterial supply areas. The ischemic infarcts were territorially distributed, matching the perfusion decreases in the supply area of the affected vessels, most of them multisite, and did not show diffuse extension typical of generalized cerebral edema. Infarcts diagnosed in the treated or affected brain areas during controls after EVT, external ventricular drain (EVD), or clipping were not categorized as vasospasm-related cerebral ischemia. As a result of vasospasm-induced massive cerebral infarcts, patients died either under regular intensive care management or after withdrawal of such measures. For these patients, the best medical treatment including analgesics was provided. All patients in Group 3 had no other relevant causes or complications that could have led to death.

Patients in Group 3 were analyzed emphasizing details considered crucial for a fatal outcome and were categorized into the following subgroups: Group 3a, “no diagnosis and treatment of vasospasm” (e.g., poor-grade aSAH and massive brain damage from the aneurysm rupture); Group 3b, “delayed diagnosis” (e.g., unconsciousness, poor conditions for TCD and TCCD, and vasospasm recognized on CT as infarcts); Group 3c, “cardiovascular complications” (e.g., identified hypotensive episodes despite vasospasm) causing death; and Group 3d, “complications attributed to the endovascular vasospasm treatment causing death”.

*Group 3a:* All patients in Group 3a suffered from aSAH with resultant vasospasm in the early phase after hemorrhage. During the initial imaging, the patients were found to have extensive ischemia with a typical distribution pattern as in the post-vasospasm state. Due to the advanced stage of cerebral ischemia, these patients were treated neither surgically nor endovascularly for the aneurysm, nor was the vasospasm treated with drugs or mechanical means. These patients did not have generalized cerebral edema or life-threatening complications other than cerebral vasospasm. In these patients, there was no further follow-up imaging and they typically died within the first few days after vasospasm onset from cerebral infarcts.

*Group 3b:* “Delayed diagnosis” means the delayed escalation of diagnosis after the detection of increased MFV. No CT scan was performed within the first two hours after TCD. In addition, there are patients with poor bone window for TCD. These patients need to receive daily CT/CTA/CTP examinations during the vasospasm phase (days 4–14), but this has been missed in some cases. This group comprises patients in whom proactive diagnostic procedures might have helped to prevent vasospasm.

*Group 3c:* “Fatal vasospasm due to cardiovascular complications” was considered when patients with vasospasm were hypotensive for more than 15 min either in the intensive care unit or during the preparatory phase before starting treatment. None of these events in Group 3c was a complication resulting from EVT. In Group 3c patients, although vasospasm was detected in time and therapy was also initiated early, the deteriorated hemodynamic situation led to extensive cerebral ischemia. This group comprises patients in whom vasospasm was diagnosed in time, but best efforts failed to prevent ischemic brain damage.

*Group 3d:* This group was reserved for patients with fatal “vasospasm-treatment complications”.

To determine the effect of an increased physician’s awareness of posthemorrhagic vasospasm, we divided the patients with non-fatal or fatal vasospasm into three equal consecutive groups. Group I, II, and III were treated between 01/2007–12/2011, 01/2012–12/2016, and 01/2017–12/2021, respectively. The frequency of delayed (fatal or non-fatal) vasospasm diagnosis in each group was counted.

All CT, CTA, CTP, MRI, MRA, and DSA images were initially evaluated blindly and assessed according to the presence of vasospasm correlates, independent of TCD values and clinical course and outcome. Patients who had vasospasm were divided into those with life-threatening vasospasm and those with non-life-threatening vasospasm based on imaging findings. We evaluated all clinical data of patients who had already died at the first clinical admission after SAH. The patients who had severe vasospasm and massive ischemia but no other life-threatening conditions such as pulmonary embolism (LAE) and multiple organ dysfunction, were defined as Group 3 (“fatal vasospasm”).

### 2.4. Statistical Analysis 

Categorical variables were presented as percentages. Continuous variables were presented as means and standard deviations. The Fisher and Mann–Whitney U-tests were used to analyze categorical and continuous variables, respectively. All statistical tests were two-sided, and *p* < 0.05 was considered significant. 

## 3. Results

During the study period, a total of 1064 patients with an aSAH were admitted to our institution. A total of 188 patients (17.6%) eventually died as a direct or indirect result of the aSAH. Only 38.4% of the patients exhibited cerebral vasospasms, with the low percentage being possibly due to early treatment with nimodipine IV from day 1. The treatment of aSAH in our clinic followed a structured SOP, approved by the senior authors.

The median time between aSAH and aneurysm treatment was 2.5 days.

Patients were classified as follows:
Group 1 (no vasospasm)656 patients(61.6%)Group 2 (non-fatal vasospasm)344 patients(32.3%)Group 3 (fatal vasospasm)64 patients(6%)

A detailed analysis of Group 3 resulted in the following categorizations:
Group 3a: no diagnosis/treatment of vasospasm31 patients (48.4%)Group 3b: delayed diagnosis26 patients (40.6%)Group 3c: fatal cardiovascular complications7 patients (10.9%)Group 3d: fatal complications of EVT for vasospasm0 patients (0%)

A total of 64 patients (median age 54 years) died from cerebral vasospasm. Cerebral vasospasm was the leading cause of death following the verification of the clinical data and neuroradiological images of all patients by the first author, under the supervision of the senior author. 

In total, 16 (25%) of these patients showed arterial hypertension, 10 (15.6%) patients were smokers, 3 (4.7%) had diabetes mellitus, 3 (4.7%) had atrial fibrillation, 3 (4.7%) had hypercholesterolemia, and only 1 patient (1.5%) had an identified positive family history of SAH as a result of a ruptured aneurysm.

Thirty-one (48.4%) of these patients died without treatment of the aneurysm or vasospasm (Group 3a). Most of these patients were female (*n* = 28, 90.3%) and they had a median Hunt and Hess grade of IV. The remaining 33 patients died due to delayed diagnosis (Group 3b; *n* = 26, 40.6%) or cardiovascular complications (Group 3c; *n* = 7, 10.9%) after successful microsurgical (*n* = 6, 18.2%) and endovascular (*n* = 27, 81.8%) aneurysm treatment. Delayed diagnosis (Group 3b) was usually concerning patients who were sedated and ventilated and could therefore not be assessed clinically, and in whom TCD did not reveal vasospasm or for whom TCD was not feasible.

The median intervals between the aSAH and both the vasospasm diagnosis and death were 6.5 days (interquartile range (IQR) = 4–7.75) and 14.33 days (IQR = 10.25–17.75), respectively. All patients were examined from day 1 using TCD. The average time taken to perform the first diagnostic imaging examination was 1.4 days (IQR = 0–2; CTA = 57.1%, MRA = 13.1%, and DSA = 19.8%). Diagnostic imaging (e.g., CTA/CTP) of possible vasospasm was triggered by suspicious TCD results, poor conditions for TCD, increasing intracranial pressure, decreasing cerebral tissue oxygen saturation, new-onset focal neurological symptoms in conscious patients, and any combination thereof. A delay in performing diagnostic imaging was usually related to the condition of the patient.

Stellate ganglion blockade was performed in 12 patients. All patients were treated with nimodipine IV or PO to prevent early vasospasm. Eighteen of these patients with vasospasm refractory to medical therapy underwent EVT. In 14 patients, IA administration of milrinone was performed; six other patients received continuous selective IA application of nimodipine. One of these patients received both EVTs while another underwent additional “stentoplasty” (i.e., mechanical vessel dilatation with a stent-retriever), as a vasospasm treatment. The average time from the rupture of the aneurysms to the performance of the initial EVT was 6.9 days (IQR = 5–7).

Illustrative case reports for each subgroup are presented below.

*Group 3a* (No Diagnosis and Treatment of Vasospasm Due to the Poor Condition of the Patient)

**Figure 1:**
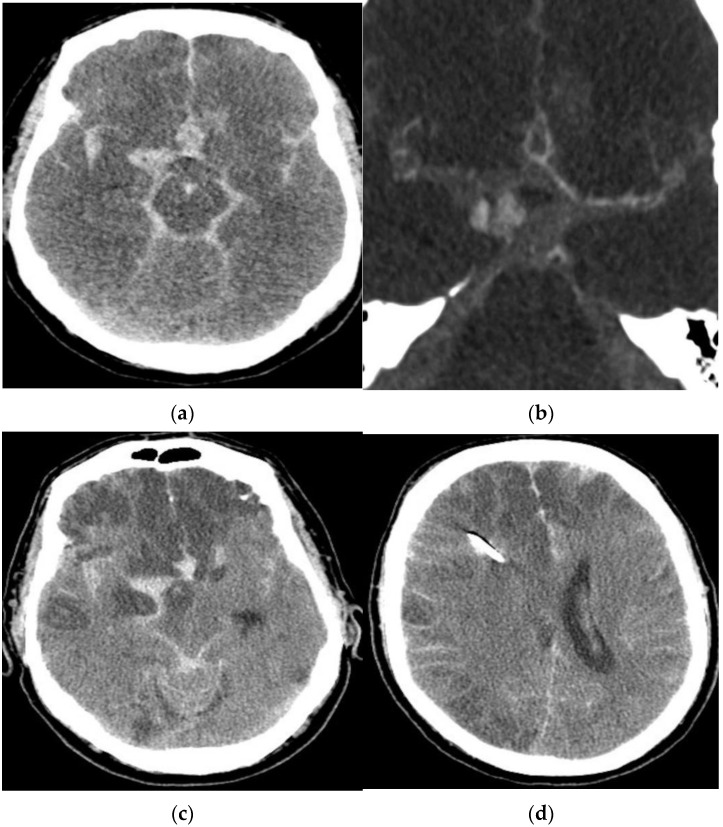
Diagnostic imaging of a 57-year-old male patient with an aSAH due to a ruptured MCA aneurysm. Computed tomography (**a**) and CTA (**b**) revealed a massive aSAH and early vasospasm. CCT after one day (**c**,**d**) showed large ischemic infarctions.

A 57-year-old man presented with a massive aSAH due to a ruptured aneurysm of the right middle cerebral artery (MCA). On admission, his clinical condition was rated as Hunt and Hess (HH) grade V. Given his poor clinical condition, conservative management was chosen. CTA on day 1 after the aSAH revealed early vasospasm. The patient passed away 1 day later. His medical history showed no evidence of a previous aSAH (Figure 1).

*Group 3b* (Delayed Diagnosis of Vasospasm)

**Figure 2:**
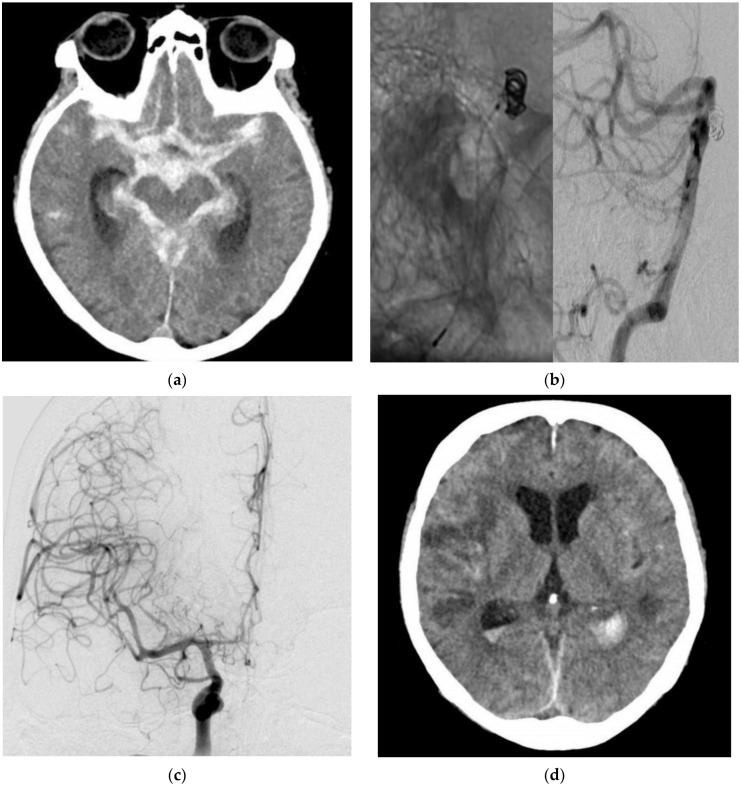
Diagnostic imaging and EVT in a 70-year-old female patient with an aSAH due to a ruptured basilar artery aneurysm. CT (**a**) showed a massive aSAH. DSA (**b**) with contrast-medium injection of the left vertebral artery (VA; lateral projection, asterisk) demonstrated the EVT with the coil mass inside the aneurysm. DSA (**c**) with contrast-medium injection of the right internal carotid artery (ICA; posterior–anterior projection) revealed massive vasospasm. Post-interventional CT (**d**) showed ischemic infarctions despite EVT.

A 70-year-old woman was diagnosed with a HH grade V aSAH. The ruptured basilar aneurysm underwent coil occlusion on the day of rupture, which was accomplished without difficulty. The patient remained intubated and sedated. Daily TCD/TCDD examinations did not show vasospasm. Routine CT/CTA on day 9 showed ischemic infarctions of both hemispheres due to vasospasm. The patient died on day 15 after the aSAH (Figure 2).

*Group 3c* (Fatal Cardiovascular Complications)

**Figure 3:**
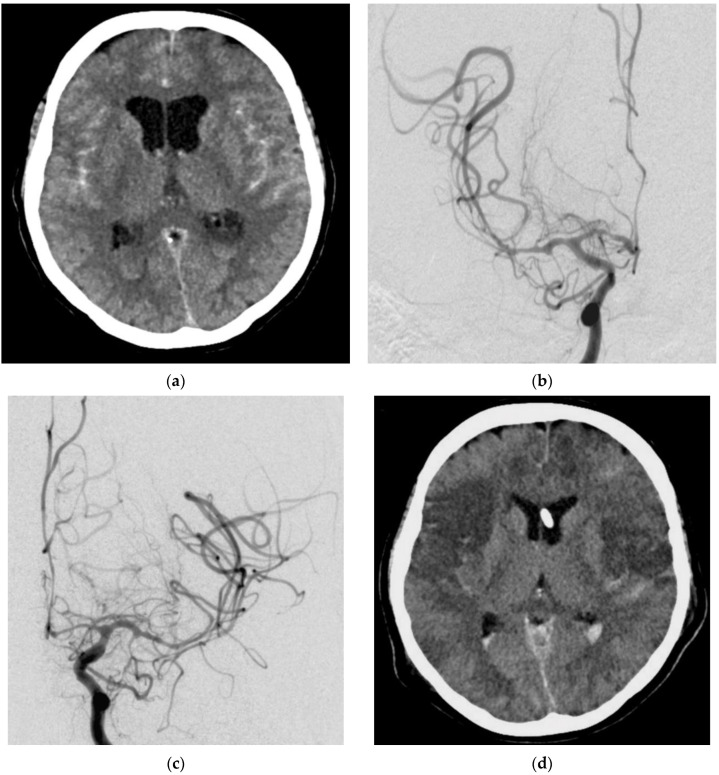
Diagnostic imaging and EVT in a 50-year-old female patient with an aSAH due to a ruptured left ICA aneurysm. Cranial CT (**a**) showed an aSAH. DSA with contrast-medium injection of both hemispheres revealed massive vasospasm (**b**,**c**). Post-interventional CT showed ischemic infarctions of both hemispheres (**d**).

A 50-year-old woman presented with a ruptured left ICA aneurysm, which was treated with a flow diverter on the day of the hemorrhage. TCD and DSA on day 4 after the aSAH revealed massive vasospasm and EVT was considered necessary. During the preparation of the catheter intervention (still on the ICU), a hypotensive episode refractory to medication occurred for 15 min with a systolic blood pressure of 90 mmHg. This hypotensive episode occurred before any therapeutic intervention. No mechanical procedure or medication administration was performed before the arterial hypotension. CT on the following day showed ischemic damage to both hemispheres and the patient died 2 days later. The ischemic infarcts in the postinterventional CT images matched the distribution of cerebral vasospasm in the previous day’s DSA images (Figure 3).

*Group 3d* (Complications of EVT for Vasospasm)

There was no death related to the EVT of vasospasm. We recorded no vessel dissection, perforation, or intracranial hemorrhage during interventions as an immediate cause of death. In one case, thrombi were dissolved after continuous selective IA application of nimodipine.

**Figure 4:**
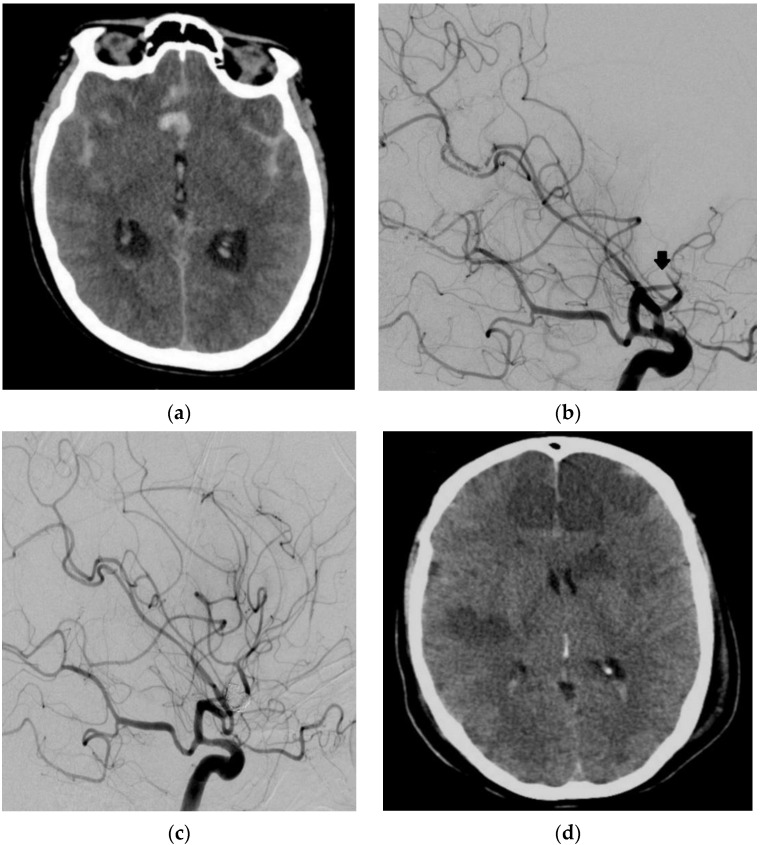
Diagnostic imaging and EVT in a 39-year-old female patient with an aSAH due to a ruptured anterior communicating artery (AcomA) aneurysm. Cranial CT showed an aSAH (**a**). DSA with a contrast-medium injection of the left ICA artery (lateral view 45°) showed an embolic occlusion of the superior trunk of the left MCA (arrow) and ACA (**b**) with the dissolution of the thrombus after infusion of rtPA (**c**). Cranial CT showed massive ischemic infarctions of both ICA and MCA supply territories (**d**).

A 39-year-old woman presented with an aSAH due to a ruptured AcomA aneurysm. The patient underwent coil occlusion one day after the admission. Routine DSA on day 8 after the aSAH showed massive vasospasm of all cerebral arteries. EVT of the vasospasm was initiated. IA administration of milrinone was performed twice, followed by continuous selective IA infusion of nimodipine. Subsequently, DSA showed embolic occlusion of the superior trunk of the left middle cerebral artery and the left anterior cerebral artery (ACA) at the transition from the A1 segment to the A2 segment. This was treated with an infusion of 5 mg recombinant tissue plasminogen activator (rtPA). No relevant brain infarct due to the thrombi was detectable on CT. Despite this, postprocedural CT showed infarctions of both hemispheres after delayed diagnosis and EVT for vasospasm. The patient passed away 8 days after the latter procedure due to the marked cerebral ischemia in multiple supply areas on both sides with typical extension after severe cerebral vasospasm (Figure 4).

**Figure 5:**
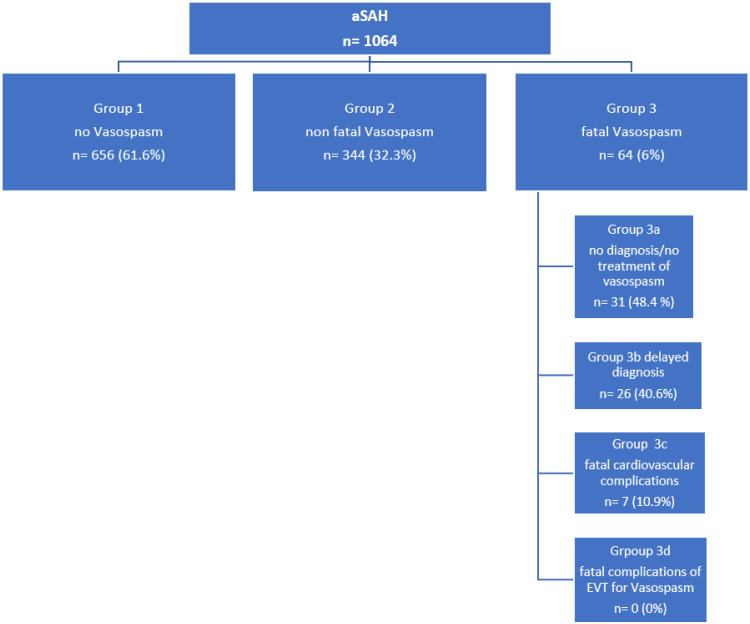
Flowchart summarizing the numbers of patients in the different subgroups.

The numbers of patients in the subgroups are summarized in Figure 5.

Among 14 identified causes of death, vasospasm was one of the main reasons for mortality after aSAH. Only four patients died primarily due to endovascular or surgical treatment complications of the aneurysm after aSAH. Of the 64 patients who died because of vasospasm, 31 were not treated by either surgical or endovascular means because of extensive brain damage from the initial aSAH. The other 33 patients passed away during hospitalization. The locations of the ruptured aneurysms are summarized in Table 1 and did not show a significant difference between the three groups. A comparison of the three groups based on sex, Hunt and Hess grade, Fisher grade, aneurysm morphology, and mode of treatment is shown in Table 2. Of the poor grade patients (Hunt and Hess IV and V), a total of 139/391(35.5%) patients died during the (sub)acute posthemorrhagic phase (90 days). In 47/139 (33.8%) patients, massive vasospasm was diagnosed and considered the primary cause of death.

The patients who eventually died from vasospasm were predominantly female. Their clinical condition was rated as Hunt and Hess grade V in >50% and as Fisher grade 4 in >90%. The different treatment techniques were not associated with fatal vasospasm. A group comparison of age and aneurysm dimensions is shown in Table 3. Age and aneurysm dimensions were irrelevant to the occurrence of fatal vasospasm. The causes of death in the 188 patients after aSAH are listed in Table 4. A multivariable analysis is presented in Table 5. 

Multivariable analysis confirmed the relevant influence of female sex (female vs. male; *p* = 0.001, adjusted OR = 1.75, 95% CI = 1.32–2.32) and increase in HH grade (4 vs. 1; *p* = 0.029, adjusted OR = 1.69, 95% CI = 1.06–2.69 and 5 vs. 1; *p* = 0.011, adjusted OR = 1.88, 95% CI = 1.15–3.06) and Fisher grade (3 vs. 1; *p* = 0.015, adjusted OR = 2.41, 95% CI = 1.18–4.88 and 4 vs. 1; *p* = 0.005, adjusted OR = 2.70, 95% CI = 1.35–5.41) when comparing fatal vasospasm with the combination of non-fatal vasospasm and no vasospasm, as well as in the comparison of fatal vasospasm with non-fatal vasospasm (female vs. male *p* = 0.001; HH 4 vs. 1 *p* = 0.029 and 5 vs. 1 *p* = 0.001; Fisher 3 vs. 1 *p* = 0.014 and 4 vs. 1 *p* = 0.005).

We performed multivariate analysis using ordered logistic regression. 

Model 1: the odds ratios in ordered logistic regression mean; the odds for fatal vasospasm versus the combination of non-fatal vasospasm and no vasospasm. 

Model 2: the odds ratios in ordered logistic regression mean; the odds for no spasm versus the combination of non-fatal vasospasm and fatal vasospasm.

A delayed imaging diagnosis of vasospasm was made in Group I (01/2007–12/2011), Group II (01/20112–12/2016), and Group III (01/2017–12/2021) in *n* = 26/154 (16.9%), *n* = 31/140 (22.1%), and *n* = 14/114 (12.2%), respectively.

While 26/64 (40.6%) patients with fatal vasospasm fell in Group b (delayed diagnosis), the same issue occurred in only 45/344 (13.1%) patients with non-fatal vasospasm. “Delayed diagnosis” remains a decisive factor in the entire vasospasm scenario. Once massive ischemic brain damage is established (and demonstrated by CT, too late for relevant treatment), any further intervention must be considered futile.

## 4. Discussion

The current data demonstrate that post-hemorrhagic cerebral vasospasm carries a significant risk of morbidity and mortality. Since the 1980s, vasospasm has been highlighted as the main cause of poor outcomes after aSAH. However, more recently, there has been a significant shift away from large-vessel vasospasm as the cause of poor outcomes, towards microvascular dysfunction and microthrombosis [30]. There are several reasons why this may have occurred, including the fact that whilst approximately 70% of patients demonstrated angiographic vasospasm following aSAH, the incidence of delayed cerebral ischemia (DCI) was around 30% [31,32]. Furthermore, there is evidence that patients with aSAH may develop DCI without angiographic large-vessel vasospasm [33,34,35,36]. Similarly, the disappointing results of drug trials using clazosentan, which significantly reduced large-vessel vasospasm but failed to improve a functional outcome or reduce mortality in aSAH patients [37,38,39,40,41], led many to believe that large-vessel vasospasm was not the root cause of the problem. In truth, it is likely that the pendulum may have swung too far away from proximal vasospasm to non-vasospastic causes of poor outcome. We believe that both conditions contribute, to a greater or lesser degree, to poor outcomes in individual patients. 

Currently, the mainstay of treatment for patients with aSAH revolves around nimodipine. For approximately three decades, triple-H therapy (hypertension, hypervolemia, and hemodilution) has been used with the hope of improving cerebral perfusion [42]. This strategy aimed to alter the different components of the Hagen–Poiseuille law to improve flow through the arterial system and thereby increase cerebral perfusion. Of the three different components of the treatment, induced hypertension appeared to be the only one that was successful in increasing cerebral perfusion [43]. However, there is now evidence to suggest that even induced hypertension may be ineffective. Gathier et al. [44] recently published the results of the first randomized controlled trial to examine the effect of induced hypertension and DCI. Although the trial aimed to recruit 240 patients, only 21 were randomized to hypertension and 20 to the control group with no induced hypertension. In the hypertensive cohort, the mean arterial pressure (MAP) over the initial 24 h was 11.1 mmHg (95% confidence interval (CI) = 7.1–15.1 mmHg) higher than that in the control group, and dropped to 5.7 mmHg (95% CI = 4.2–8.5 mmHg) over 72 h. A poor outcome (90-days modified Rankin scale (mRS) score = 4–6) was seen in 12 of the 21 (57%) patients in the treatment group, and in 8 of the 20 (40%) patients in the control group. In the treatment arm, induced hypertension resulted in an adjusted risk ratio (RR) of 1.0. In the initial 24 h of symptoms, 18 patients showed a clinical improvement (*n* = 12, 67% in the treatment cohort; and *n* = 6, 33% in the control group), defined as any improvement in the Glasgow coma score or of focal deficits. However, 5 of the 12 patients with initial improvement in the treatment arm had a poor outcome, and none of the six patients with initial improvement in the control arm had a poor outcome at 3 months. Serious adverse events occurred in 11 of the 21 patients (52%) in the treatment cohort, and 5 of the 20 (25%) patients in the control group (RR = 2.1, 95% CI = 0.9–5.0). This study highlighted that immediate clinical improvement may not result in long-term functional improvement and that hypertension carries a significant risk of associated complications. Mathematical modeling has previously been used to assess the effect of induced hypertension in vasospastic cerebral arteries whilst simultaneously modeling different arterial configurations (e.g., the presence of an AcomA), vasospasm affecting different vessels, and differing degrees of vasospasm. The results of such modeling, perhaps not surprisingly, showed marked differences in the ability of hypertension to improve perfusion [45] and may account for the limited clinical response reported by Gathier et al. [44].

Jabbarli et al. [46] investigated the efficacy of vasospasm management on the outcome. They compared two cohorts from two institutions, which included 1057 patients treated between 2005 and 2012. There were no differences in baseline demographics, including age, gender, Hunt and Hess grade, acute hydrocephalus, treatment modality, infections, and radiological features (aneurysm location and Fisher grades). In both cohorts, patients underwent daily TCD ultrasonography. Conservative management included oral nimodipine (360 mg/day) and maintenance of normovolemia for 3 weeks. The MAP was kept at ≥70 mmHg using colloids or inotropes. The subsequent management varied between the two cohorts: a mean flow velocity of >120 cm/s was used in cohort A, while an MFV of 160 cm/s was used in cohort B, as the cut-off for vasospasm. Initiation of induced hypertension varied between the two groups: in cohort A, at the time of diagnosis of vasospasm, the MAP was raised to 80 mmHg; while, in cohort B, it was raised in a stepwise manner to 110 mmHg. With respect to the initiation of EVT, patients in cohort A underwent immediate DSA for EVT when there was any suspicion of delayed ischemic neurologic deficit (DIND), regardless of the TCD values or the development of TCD vasospasm in patients with limited neurological assessability. By contrast, patients in cohort B underwent EVT if DIND/DCI persisted despite the escalation of induced hypertension within 2–4 h, or in cases of TCD vasospasm gradually exceeding the aforementioned cut-off during two consecutive days.

An EVT was indicated if there was ≥50% arterial narrowing compared to the initial DSA or the contralateral vessel(s). As a first line EVT, pharmacological vessel dilatation was performed, and in some cases, balloon angioplasty was employed in proximal branches. Repeat procedures were carried out as necessary. In cohort A, 24.4% of the patients (*n* = 121/495) underwent an initial EVT on day 6 ± 3.64, and in cohort B, 14.4% (*n* = 81/562) underwent EVT on day 8.9 ± 4.78 with statistically significant differences in the rate (*p* < 0.0001, odds ratio (OR) = 1.92, 95% CI = 1.41–2.63) and the timing (*p* < 0.0001) of treatment. As predicted, there was also a difference in the indication for treatment, with 66.1% of patients undergoing EVT in cohort A based on TCD without evidence of DIND (66.1% vs. 42%, *p* = 0.0001), and a correspondingly lower proportion of patients undergoing treatment of EVT with evidence of DIND. The rate of DCI was lower in cohort A (20.8% vs. 29%, *p* = 0.0023, OR = 0.64, 95% CI = 0.48–0.85), as confirmed by multivariate analysis (*p* = 0.001, adjusted OR = 0.59, 95% CI = 0.44–0.8). The rates of DCI were higher in patients undergoing EVT for DIND compared to those undergoing EVT solely due to TCD measurements (64% vs. 44.7%, *p* = 0.0277). The rate of unfavorable outcome after SAH was also lower in cohort A (44% vs. 56%, *p* = 0.0404) and remained significant in multivariate analysis (*p* < 0.0001, adjusted OR = 0.55, 95% CI = 0.4–0.77). This study demonstrated that aggressive treatment may result in better functional outcomes and that early identification and treatment of vasospasm may improve outcomes. Similarly, it confirmed that hypertension and delays in performing EVT may adversely affect the outcome, as suggested by Gathier et al. [44]. 

Given these results, it is imperative to identify patients with vasospasm as early as possible and to instigate EVT in the treatment of large-vessel occlusions and strokes. Imaging methods that can be performed at the bedside would be ideal for these purposes. One of the principal issues with TCD is that it is highly user-dependent and time-consuming. Imaging via CTA and CTP provide alternatives, albeit with significant logistic efforts and radiation exposure. Automated systems, such as robotic TCD or continuous near-infra-red spectroscopy (NIRS), could provide alternative means of solving the problem of early detection. Recently, Esmaeeli et al. [9] reported their experience of using a robotic TCD system to detect vasospasm in MCA and ACA. They demonstrated that robotically-detected MFVs were comparable to manual TCDs in the MCA (concordance correlation coefficient (CCC) = 0.83, 95% CI = 0.42–0.96, *p* = 0.001) but fared poorly in the ACA (CCC = 0.26, 95% CI = (−0.01)–0.71, *p* = 0.26). Despite being a preliminary study, this demonstrated that automated systems may play a role in the detection and management of vasospasm in the future. An ongoing trial using NIRS to identify patients with early vasospasm is currently underway (ClinicalTrials.gov Identifier: NCT04042571) and, if successful, may provide a further non-invasive and automated way to detect cerebral vasospasm. In our own series, 40.6% (*n* = 26/64) of patients were in Group 3b, in which diagnosis and/or treatment were delayed. It is certain that early accurate diagnosis, just as in stroke due to large-vessel occlusion, will be crucial for improving the outcome of these patients. Other centers have adopted routine imaging protocols at set time points to identify patients with cerebral vasospasm. Chen et al. [23] recently published their results on the use of the Scepter XC balloon catheter (MicroVention) with simultaneous nimodipine infusion. In this study, all patients with aSAH underwent bedside TCD; in patients with suspected vasospasm, further imaging with CTA and CTP was used to confirm the diagnosis. For patients in which TCD could not be adequately performed, for example, due to poor temporal bone windows, CTA and CTP were routinely utilized on day 7 post-ictus. Invasive treatment was performed for patients who did not respond to hypertensive treatment. This involved either IA nimodipine alone if the vasospasm was <25%, or Scepter XC balloon angioplasty if the vasospasm was more severe or failed to respond after IA nimodipine infusion. The authors identified 50 patients (mean age = 50 years, range = 28–68 years; 76% female) who underwent balloon angioplasty and nimodipine infusion. In most patients, angioplasty involved the ICA, M1, or M2 segments. The authors reported no cases of vessel rupture, dissection, or thromboembolism. Angiographic-image improvement was seen in 100% of cases, with 94% of patients exhibiting symptomatic improvement and 82% of patients achieving a good functional outcome (mRS ≤ 2) at 90 days. We attribute these promising results, at least partly, to the use of routine advanced imaging to detect vasospasm. In our series, the median interval between ictus and cerebral vasospasm was 6.5 days. The routine use of CTA and CTP on day 7 may have helped identify a greater number of patients amenable to treatment and hence reduced the number of patients in Groups 3a and 3b. 

Any treatment should be targeted to the affected vessels to avoid creating a “steal” phenomenon. Levitt et al. [47] published their experience with real-time assessment of angiographic perfusion after EVT for cerebral vasospasm. They demonstrated that cerebral blood flow was improved in all the treated segments but deteriorated in untreated segments (e.g., balloon angioplasty in the M1 segment could result in flow improvement in the MCA territory but could worsen flow in a vasospastic ACA). Shimamura et al. [48] observed that the non-selective injection of vasodilators produced a relatively greater improvement of flow in a non-spastic artery and, hence, resulted in an iatrogenic steal phenomenon. These results were expected, given that IA vasodilators travel with the greatest flow and therefore preferentially move away from the most spastic vessels if they are not delivered to the site of spasm. Similarly, distal vasospasm may be best treated using medication, whereas proximal vasospasm may be better treated using mechanical means. In this regard, an individually tailored approach dependent upon the angiographic findings should be adopted. Based on previous studies, it may also be beneficial to target the proximal vasospasm prior to the injection of vasodilators, as this may increase the effectiveness of any mechanical treatment [27]. 

Earlier detection and intensive monitoring are only the initial steps in the paradigm shift that is needed for treatment to prevent poor outcomes following aSAH [49]. Adequate EVT is also necessary with a variety of treatment options, ranging from balloon angioplasty [19,20,21,22,23,24] and stentoplasty [25,26,27,28,29] to IA medicinal vessel dilatation, in addition to combined approaches. As is the case with large-vessel occlusive stroke, there has been a move towards imaging-based decision making, which may be of great importance in cerebral vasospasm as multiple factors may be involved. 

## 5. Conclusions

This study shows that cerebral vasospasm is one of the most significant causes of death following aSAH. Despite the development of examination and treatment routines, fatal vasospasm is still not always preventable. This study showed a group of patients in whom the fatal outcome was unavoidable due to the early occurrence after aSAH. However, in a large proportion of patients, the fatal outcome of vasospasm remains preventable by early diagnosis and treatment and by strict stabilization of the circulation during the vasospasm phase.

In our clinical practice, we aim to perform daily monitoring of intracranial vessels by CTA or MRA scans and permanent cardiovascular monitoring in patients after aSAH. Endovascular treatment of vasospasm has good safety margins, without fatal complications in our long-term study. For the time being, early detection of vasospasm remains the prerequisite for vasospasm treatment to be effective against life-threatening brain ischemia.

There is an ongoing dilemma in vasospasm management. The enormous logistic and financial burden of one- or two-week daily CTA would be better justified if there were unambiguous proof of the superiority of endovascular over medicinal vasospasm treatment, which is pending thus far. The most efficacious treatment, however, is futile if applied too late (i.e., to already infarcted brain tissue). However, this again requires early detection of vasospasm before brain infarcts occur. The only mitigating factor would be a safe and reliable prophylactic procedure, applied early that is able to prevent vasospasm during the later course. 

The management of patients with aSAH has become more complex, far beyond the immediate aneurysm occlusion. Management requires a multidisciplinary effort between neurosurgery, neuroradiology, and neurocritical care and should be carried out exclusively in specialized tertiary neurovascular centers. Overcoming the fatalistic attitude against vasospasm and proactive treatment of this condition is the current challenge in the treatment of aSAH.

## Figures and Tables

**Table 1:** Location of aneurysms in Group 1 (no vasospasm), 2 (non-fatal vasospasm), and 3 (fatal vasospasm).

	Group 1 *n* = 661	Group 2 *n* = 338	Group 3 *n* = 64
AchoA	6 (0.9%)	4 (1.2%)	2 (3.1%)
AcomA	224 (33.9%)	113 (33.4%)	20 (31.3%)
AICA	0 (0%)	1 (0.3%)	1 (1.6%)
Basilar artery bifurcation	43 (6.5%)	15 (4.5%)	8 (12.5%)
Basilar artery trunk	21 (3.2%)	2 (0.6%)	1 (1.6%)
Callosomarginal/pericallosal artery	35 (5.3%)	15 (4.4%)	0 (0%)
Carotid bifurcation	11 (1.7%)	5 (1.5%)	2 (3.1%)
MCA bifurcation	119 (18%)	75 (22.2%)	13 (20.3%)
MCA, M1	6 (0.9%)	5 (1.5%)	0 (0%)
MCA, M2	8 (1.2%)	6 (1.8%)	1 (1.6%)
Paraophthalmic ICA	21 (3.2%)	13 (3.8%)	1 (1.6%)
PCA, P1	8 (1.3%)	3 (0.9%)	1 (1.6%)
PcomA	90 (13.6%)	55 (16.3%)	13 (20.3%)
PICA	33 (5.0%)	9 (2.7%)	0 (0%)
SCA	7 (1.1%)	2 (0.6%)	0 (0%)
VA junction	2 (0.3%)	0 (0%)	0 (0%)
VA, V4	12 (1.8%)	9 (2.7%)	1 (1.6%)

Abbreviations: AchoA: anterior choroidal artery; AcomA: anterior communicating artery; AICA: anterior inferior cerebellar artery; ICA: internal carotid artery; MCA: middle cerebral artery; PCA: posterior cerebral artery; PcomA: posterior communicating artery; PICA: posterior inferior cerebellar artery; SCA: superior cerebellar artery; VA: vertebral artery.

**Table 2:** Comparison of sex, Hunt and Hess grade, Fisher grade, aneurysm morphology, and treatment technique in Group 1 (no vasospasm), 2 (non-fatal vasospasm), and 3 (fatal vasospasm).

		Group 1 *n* = 661	Group 2 *n* = 338	Group 3 *n* = 64	*p*-Value	
Sex	F	410 (62.0%)	234 (69.2%)	53 (82.8%)	1 vs. 2	0.025
	M	251 (38.0%)	104 (30.8%)	11 (17.2%)	2 vs. 3	0.034
					3 vs. 1	0.001
Hunt and Hess grade	1	133 (20.1%)	60 (17.8%)	2 (3.1%)	1 vs. 2	0.033
	2	175 (26.5%)	78 (23.1%)	3 (4.7%)	2 vs. 3	<0.001
	3	126 (19.1%)	83 (24.6%)	12 (18.8%)	3 vs. 1	<0.001
	4	86 (13.0%)	60 (17.8%)	11 (17.2%)		
	5	141 (21.3%)	57 (16.9%)	36 (56.3%)		
Fisher grade	1	46 (7.0%)	10 (3.0%)	1 (1.6%)	1 vs. 2	0.054
	2	72 (10.9%)	32 (9.5%)	0 (0%)	2 vs. 3	<0.001
	3	121 (18.3%)	70 (20.7%)	4 (6.3%)	3 vs. 1	<0.001
	4	422 (63.9%)	226 (66.9%)	59 (92.2%)		
Aneurysm morphology		1 (*n* = 644)	2 (*n* = 337)	3 (*n* = 57)		
	Saccular	594 (92.3%)	315 (93.5%)	51 (89.6%)	1 vs. 2	0.014
	Dissecting	31 (4.8%)	16 (4.7%)	5 (8.8%)	2 vs. 3	0.366
	Fusiform	13 (2.0%)	2 (0.6%)	0 (0%)	3 vs. 1	0.529
	Blister	6 (0.9%)	2 (0.6%)	1 (1.8%)		
Treatment		1 (*n* = 600)	2 (*n* = 336)	3 (*n* = 37)		
	Clipping	123 (20.5%)	89 (26.5%)	8 (21.6%)	1 vs. 2	0.442
	Coiling	352 (58.7%)	182 (54.2%)	20 (54.1%)	2 vs. 3	0.370
	Flow diverter	34 (5.7%)	20 (6.0%)	2 (5.4%)	3 vs. 1	0.332
	pCONUS-assisted coiling	38 (6.3%)	21 (6.3%)	4 (10.8%)		
	Stent-assisted coiling	26 (4.3%)	13 (3.9%)	0 (0%)		
	Parent vessel occlusion	13 (2.2%)	7 (2.1%)	3 (8.1%)		
	Liquid embolic agent	3 (0.5%)	0 (0%)	0 (0%)		
	Flow diverter-assisted coiling	11 (1.8%)	4 (1.2%)	0 (0%)		

**Table 3:** Comparison of age and aneurysm size in Group 1 (no vasospasm), 2 (non-fatal vasospasm), and 3 (fatal vasospasm).

	Group	n	Median	Min–Max	Comparison *p*-Value	
Age						
	1	661	57.7	0.3–95.9	1 vs. 2	<0.001
	2	338	52.0	14.3–88.6	2 vs. 3	0.069
	3	64	53.5	26.6–91.2	3 vs. 1	0.276
Aneurysm depth						
	1	628	5.6	1.0–42.0	1 vs. 2	0.701
	2	332	5.8	1.0–31.4	2 vs. 3	0.151
	3	57	6.5	1.0–24.0	3 vs. 1	0.219
Aneurysm width						
	1	628	4.0	0.7–40.0	1 vs. 2	0.136
	2	332	4.0	1.0–25.0	2 vs. 3	0.070
	3	57	5.0	1.0–16.0	3 vs. 1	0.250

**Table 4:** Causes of death after aSAH.

Causes of Death	N= 188
Endovascular treatment complication	*n* = 1 (0.5 %)
Surgical treatment complication	*n* = 3 (1.6 %)
Vasospasm	*n* = 64 (34%)
Rebleeding	*n* = 25 (13.3%)
Brain herniation	*n* = 49 (26.1%)
Multiple organ dysfunction	*n* = 19 (10.1%)
Pulmonary embolism	*n* = 3 (1.6%)
Acute respiratory distress syndrome	*n* = 12 (6.4%)
Myocardial infarction/cardioplegia	*n* = 7 (3.7%)
Other reasons (endocarditis, bleeding of esophageal varices, traumatic injuries)	*n* = 5 (2.6%)

**Table 5:** (**a**) Multivariate analysis (no vasospasm, non-fatal vasospasm, fatal vasospasm) proportional odds assumption. (**b**) Multivariate analysis (generalized ordered logistic regression). Comparison of non-fatal vasospasm versus no vasospasm and fatal vasospasm versus non-fatal vasospasm.

		Model 1	*p*-Value	Model 2	*p*-Value
		OR (95%-CI)		OR (95%-CI)	
(a)					
Sex	Female vs. male	1.75 (1.32; 2.32)	<0.001	1.74 (1.31; 2.31)	<0.001
HH grade	2 vs. 1	0.97 (0.65; 1.45)	0.873	0.96 (0.65; 1.44)	0.854
	3 vs. 1	1.47 (0.96; 2.24)	0.075	1.46 (0.96; 2.23)	0.078
	4 vs. 1	1.69 (1.06; 2.69)	0.029	1.69 (1.06; 2.70)	0.028
	5 vs. 1	1.88 (1.15; 3.06)	0.011	1.89 (1.17; 3.06)	0.010
Fisher grade	2 vs. 1	1.70 (0.79; 3.64)	0.174	1.70 (0.79; 3.65)	0.175
	3 vs. 1	2.41 (1.18; 4.88)	0.015	2.39 (1.18; 4.86)	0.016
	4 vs. 1	2.70 (1.35; 5.41)	0.005	2.70 (1.35; 5.42)	0.005
Aneurysm morphology	Regular vs. non-regular	1.60 (1.00; 2.56)	0.049	1.61 (1.01; 2.58)	0.046
Age at aSAB		0.97 (0.96; 0.98)	<0.001	0.97 (0.96; 0.98)	<0.001
**(b)**					
Sex	Female vs. male	1.71 (1.29; 2.27)	<0.001	1.71 (1.29; 2.27)	<0.001
HH grade	2 vs. 1	0.97 (0.64; 1.47)	0.871	0.97 (0.64; 1.47)	0.871
	3 vs. 1	1.57 (1.00; 2.45)	0.049	1.57 (1.00; 2.45)	0.049
	4 vs. 1	1.73 (1.06; 2.84)	0.029	1.73 (1.06; 2.84)	0.029
	5 vs. 1	1.50 (0.93; 2.40)	0.095	5.67 (3.00; 10.72)	<0.001
Fisher grade	2 vs. 1	1.79 (0.81; 3.99)	0.152	0.00 (0.00; 0.00)	<0.001
	3 vs. 1	2.51 (1.21; 5.24)	0.014	2.51 (1.21; 5.24)	0.014
	4 vs. 1	2.83 (1.37; 5.82)	0.005	2.83 (1.37; 5.82)	0.005
Aneurysm morphology	Regular vs. non-regular	1.69 (1.09; 2.63)	0.019	1.69 (1.09; 2.63)	0.019
Age at aSAB		0.96 (0.95; 0.97)	<0.001	0.99 (0.97; 1.02)	0.650

## Data Availability

The data presented in this study are available on request from the first author. The data are not publicly available due to patient privacy protection.

## Abbreviations

ACA: anterior cerebral artery; AcomA: anterior communicating artery; aSAH: aneurysmal subarachnoid hemorrhage; BA: basilar artery; CCC: concordance correlation co-efficient; CI: confidence interval; CSF: cerebrospinal fluid; CT: computed tomography; CTA: computed tomography angiography; CTP: computed tomography perfusion; DCI: delayed cerebral ischemia; DIND: delayed ischemic neurologic deficit; DSA: digital subtraction angiography; EVD: external ventricular drain; EVT: endovascular vasospasm treatment; HH: Hunt and Hess; IA: intra-arterial; ICA: internal carotid artery; IQR: interquartile range; IV: intravenous; LAE: pulmonary embolism; MAP: mean arterial pressure; MCA: middle cerebral artery; MFV: mean flow velocity; MRA: magnetic resonance angiography; MRI: magnetic resonance imaging; mRS: modified Rankin scale; NIRS: near infra-red spectroscopy; OR, odds ratio; PO: per os; RR: risk ratio; rtPA, recombinant tissue plasminogen activator; SOP: standard operating procedure; TCCD: transcranial color-coded Doppler; TCD: transcranial Doppler sonography; VA: vertebral artery.
